# When the geopolitical threat of China stokes bias against Asian Americans

**DOI:** 10.1073/pnas.2217950119

**Published:** 2022-12-07

**Authors:** Jennifer Lee

**Affiliations:** ^a^Department of Sociology, Columbia University, Knox Hall, New York, NY 10027

This year marks the 40th anniversary of the brutal murder of Vincent Chin, a 27-y-old Chinese American draftsman who was bludgeoned to death with a baseball bat by two White autoworkers in Detroit, Michigan. At the time of Chin’s killing, Japanese car imports were rising, Detroit’s auto industry was collapsing, and the United States was in the midst of a national recession. “It’s because of you little mother*ckers that we’re out of work,” said one of the White men before killing Chin (1). It did not matter that Chin was not Japanese nor did it matter that he did not work in the auto industry. Chin was Asian, which was enough to reduce him to a foreign “other” who unjustly consumes opportunities that should go to “real” Americans. Japan was perceived as a national adversary and an economic threat to the United States in the latter half of the 20th century, but China has occupied this space in the beginning of the 21st century. Four decades after the killing of Vincent Chin, Qian He and Yu Xie raise the question: Does the geopolitical threat of China stoke bias against Asian Americans? Drawing on novel experiments and survey questions that measure Americans’ assessment of the multidimensional threat of China, the authors deftly address this question in their groundbreaking paper, “The Moral Filter of Patriotic Prejudice: How Americans View Chinese in the Era of COVID-19” (2).

Qian He and Yu Xie raise the question, does the geopolitical threat of China stoke bias against Asian Americans?

## A Surge in Anti-Asian Violence, Hate Crimes, and Bias

He and Xie’s project has assumed a moral urgency amid a surge in anti-Asian violence, hate crimes, and bias since the onset of COVID-19. One in 6 Asian American adults has been a victim of a hate crime in 2021, up from 1 in 8 in 2020. By the first 3 mo of 2022, the figure had already reached 1 in twelve (3). In California, home to 6 million Asian Americans, Native Hawaiians, and Pacific Islanders, more than 1 in 4 has either experienced or witnessed an anti-Asian hate incident (4). For many observers, the rise in anti-Asian bias is a result of Trump’s repeated reference to the coronavirus as the “Chinese virus,” the “Wuhan virus,” and “kung flu”—blatantly disregarding the World Health Organization’s advice of not attaching locations or ethnicity to disease to avoid stigmatization. Words have consequences. Just 3 wk of “China virus” rhetoric in the media offset 13 prior years of declines in anti-Asian bias; Americans exposed to the rhetoric were more likely to perceive Asian Americans as foreign and un-American, with politically conservative Americans most affected by the rhetoric (5).

But it is a mistake to reduce COVID-related violence and bias to Trump’s rhetoric alone. Even before the pandemic, hate crimes against Asians during the Trump administration increased 31% between 2016 and 2018 (6). And even in a new administration, they continue to spike. While anti-Asian bias is not universal (7), 1 in 5 Americans now believe that Asian Americans are at least partly responsible for COVID-19, up from 1 in 10 in 2021. Non–Asian Americans are also more likely to believe that referring to the coronavirus as the Chinese virus and Wuhan virus is appropriate. And 1 in 3 Americans believe that Asians are more loyal to their country of origin than to the United States, up from 1 in 5 in 2021 (8).

## The Geopolitical Threat of China and Bias against Asians

While social scientists have historicized this moment by linking it to an underrecognized legacy of anti-Asian bias, violence, and exclusion (9), He and Xie turn our attention to the global present. Given China’s rapid economic ascent in the context of tenuous United States–China relations, the authors examine whether the perceived threat of China to the United States incites bias against Asian Americans. Furthermore, they examine whether Americans make distinctions among Asians in their bias against them or whether they relegate all Asians to the status of the foreign “other.” More specifically, do Americans distinguish among East Asians in the United States, Chinese in China, and Japanese in Japan? The comparison is deliberate: China has long displaced Japan as the economic adversary to the United States. The authors do not stop their inquiry there. Recognizing the extreme political polarization of the United States, He and Xie examine how political orientation affects anti-Asian bias.

Joining research on the stereotype content model with the literature on group threat, the authors assess whether and how the threat of China affects Americans’ perceptions of East Asians on four dimensions: trustworthiness, morality, competence, and warmth. The latter two dimensions form the core of the stereotype content model: How a group is perceived on competence and warmth determine how they are received and treated by others. Prior research shows that Asians are persistently perceived as competent but cold, generating feelings of envy and distrust (10, 11). The authors’ inclusion of trustworthiness and morality is both novel and germane to understanding anti-Asian bias in particular. Questions about the trustworthiness and morality of Asians date back more than 150 y, forming the foundation to a legacy of Asian exclusion from US citizenship and immigration.

Beginning with the Page Act of 1875 that banned women from any “Oriental country” on the presumption that they were immoral prostitutes and vectors of contagious diseases, laws of exclusion soon expanded to Chinese male laborers with the 1882 Chinese Exclusion Act. Excluded from immigrating, Chinese were deemed foreign economic competitors who threatened the livelihoods, health, and morals of working-class White families (11–14). Chinese exclusion was not repealed until 1943, and America’s long-standing policy of restricting immigrants from non–European countries was not abolished until 1965 when the Congress passed the Immigration and Nationality Act.

Americans may not know about the origins of Asian exclusion to be intimately familiar with the pernicious stereotypes of Asians—they are economic competitors, indelibly foreign, untrustworthy, and disloyal (15). He and Xie push the research forward by examining whether the “threat of China” affects Americans’ perceptions of East Asians in the United States. Using these ratings as the baseline, the authors then assess whether the threat of China affects Americans’ perceptions of Chinese and Japanese in their respective countries on the same measures: competence, warmth, morality, and trustworthiness.

## Bias against Chinese Nationals

He and Xie’s findings are novel. While prior research shows that Americans make distinctions among Asians and draw a sharp boundary between East and South Asians (16), He and Xie find that Americans also make distinctions among East Asians. They express the strongest bias against Chinese in China, and the bias is most pronounced among those who perceive China as a threat to the United States. The relationship is not only significant but also causal. Moreover, it is on the dimensions of trustworthiness, morality, and warmth that Americans rate Chinese in China most poorly, while they rate Japanese in Japan similarly to East Asians in the United States.

The authors make three critical interventions with their research. First, the difference in ratings between Chinese and Japanese in their respective countries underscores that anti-Asian bias cannot be reduced to blanketed xenophobia against Asians or to the perceived foreignness of East Asians. Americans distinguish not only among East Asians but also differentiate between East Asians in the United States and those in Asia. Not all Americans, however, make these distinctions.

Second, through contextual analyses, He and Xie also find that as the proportion of residents in a county that voted for Trump in 2016 increases, perceptions of trustworthiness and morality decline for all three East Asian groups, with the steepest decline for Asian Americans. Another way to think about this is that the increased bias against Asian Americans among Trump supporters is extreme enough to level anti-Asian bias among all Asian groups so that there are no longer significant differences in perceptions among them. Americans living in Republican counties rate both East Asian Americans and Chinese in China equally poorly on trustworthiness and morality. By contrast, residents in the predominantly Democrat-voting counties viewed Asian Americans more favorably than Chinese in China and also viewed the Chinese in China significantly more negatively than Asians in the United States and Japanese in Japan. These findings help explain not only why Trump’s racist and xenophobic reference of the coronavirus as the Chinese virus was so effective in igniting anti-Asian bias among his political constituents but also why Asian Americans were both outraged and threatened by Trump’s racist rhetoric (17, 18).

The third novel finding from He and Xie’s study is that the bias against Chinese in China is along specific dimensions—trustworthiness, morality, and warmth. That the ratings for competence are the most stable across all Asian groups indicates the resilience of perceived competence as a status characteristic of East Asians regardless of national origin.

## Consequences of Hypercompetence, Hypowarmth, Low Trust, and Presumed Immorality

The combination of hypercompetence, hypowarmth, low trust, and presumed immorality places Asians in the United States in a tenuous position, especially in the context of Americans’ widely shared view that China is an adversary and a national threat. One notable consequence is that Chinese American scientists have come under federal scrutiny for their associations with China under the 2018 China Initiative. Launched by then-President Trump’s administration to thwart espionage by the Chinese government, the China Initiative resulted in charges against about two dozen academic scientists, most of whom are of Chinese origin. But rather than focusing on espionage, the China Initiative targeted Chinese scientists in the United States who had any affiliations with China. Despite the failed prosecutions, including that of Professor Gang Chen at MIT, the accusations and lengthy investigations have upended the careers and lives of  many of these pioneering scientists. The China Initiative also produced a chilling effect for United States–China scientific collaborations (19). After 4 y of investigations, the Department of Justice—under a new administration—announced on February 23, 2022, that the China Initiative would come to a formal end. Apart from renaming the program, the Department of Justice has broadened its strategy to counter nation-state threats to the United States.

While a critical step, the end of the China Initiative will neither end bias against Chinese nationals nor will it end anti-Asian bias. As He and Xie perceptively note, “nationality-based prejudices against Chinese today are reminiscent of Americans’ low trust of and antagonistic attitudes toward prospering Japan in the 1980s.” Vincent Chin was a victim of this prejudice four decades ago, and Asian Americans—including Chinese scientists—are among the victims today. That Vincent Chin was not Japanese is a reminder how nationality-based prejudices can affect Asians in the United States. The surge in violence and bias against Asian Americans during the pandemic underscores this reality. The widely shared view among Americans that China is a national adversary and domestic threat suggests that bias against Chinese nationals will endure after the COVID-19 pandemic and may spill over to East Asians in the United States regardless of national origin, nativity, or citizenship.
